# MarineMetagenomeDB: a public repository for curated and standardized metadata for marine metagenomes

**DOI:** 10.1186/s40793-022-00449-7

**Published:** 2022-11-18

**Authors:** Muhammad Kabiru Nata’ala, Anderson P. Avila Santos, Jonas Coelho Kasmanas, Alexander Bartholomäus, João Pedro Saraiva, Sandra Godinho Silva, Tina Keller-Costa, Rodrigo Costa, Newton C. M. Gomes, André Carlos Ponce de Leon Ferreira de Carvalho, Peter F. Stadler, Danilo Sipoli Sanches, Ulisses Nunes da Rocha

**Affiliations:** 1 Department of Environmental Microbiology, Helmholtz Centre for Environmental Research – UFZ GmbH, 04318 Leipzig, Saxony Germany; 2 Department of Computer Science and Interdisciplinary Centre of Bioinformatics, University of Leipzig, 04107 Leipzig, Saxony Germany; 3 Institute of Mathematics and Computer Sciences, University of Sao Paulo, São Carlos, Brazil; 4 Section 3.7 Geomicrobiology, GFZ German Research Centre for Geosciences, 14473 Telegrafenberg, Potsdam Germany; 5 Department of Bioengineering and Institute for Bioengineering and Biosciences, Instituto Superior Técnico, Universidade de Lisboa, 1049-001 Lisbon, Portugal; 6 Department of Biology and Centre for Environmental and Marine Studies (CESAM), University of Aveiro, 3810-193 Aveiro, Portugal; 7 Federal University of Technology - Paraná (UTFPR), Cornélio Procópio, Brazil

**Keywords:** Marine microbiomes, Metagenomics, Microbial ecology, Metadata, Database

## Abstract

**Background:**

Metagenomics is an expanding field within microbial ecology, microbiology, and related disciplines. The number of metagenomes deposited in major public repositories such as Sequence Read Archive (SRA) and Metagenomic Rapid Annotations using Subsystems Technology (MG-RAST) is rising exponentially. However, data mining and interpretation can be challenging due to mis-annotated and misleading metadata entries. In this study, we describe the Marine Metagenome Metadata Database (MarineMetagenomeDB) to help researchers identify marine metagenomes of interest for re-analysis and meta-analysis. To this end, we have manually curated the associated metadata of several thousands of microbial metagenomes currently deposited at SRA and MG-RAST.

**Results:**

In total, 125 terms were curated according to 17 different classes (e.g., biome, material, oceanic zone, geographic feature and oceanographic phenomena). Other standardized features include sample attributes (e.g., salinity, depth), sample location (e.g., latitude, longitude), and sequencing features (e.g., sequencing platform, sequence count). MarineMetagenomeDB version 1.0 contains 11,449 marine metagenomes from SRA and MG-RAST distributed across all oceans and several seas. Most samples were sequenced using Illumina sequencing technology (84.33%). More than 55% of the samples were collected from the Pacific and the Atlantic Oceans. About 40% of the samples had their biomes assigned as ‘ocean’. The ‘Quick Search’ and ‘Advanced Search’ tabs allow users to use different filters to select samples of interest dynamically in the web app. The interactive map allows the visualization of samples based on their location on the world map. The web app is also equipped with a novel download tool (on both Windows and Linux operating systems), that allows easy download of raw sequence data of selected samples from their respective repositories. As a use case, we demonstrated how to use the MarineMetagenomeDB web app to select estuarine metagenomes for potential large-scale microbial biogeography studies.

**Conclusion:**

The MarineMetagenomeDB is a powerful resource for non-bioinformaticians to find marine metagenome samples with curated metadata and stimulate meta-studies involving marine microbiomes. Our user-friendly web app is publicly available at https://webapp.ufz.de/marmdb/.

**Supplementary Information:**

The online version contains supplementary material available at 10.1186/s40793-022-00449-7.

## Background

Metagenomics is a high throughput sequencing-based method that provides the opportunity to study microbial communities' structure and functional dynamics in the environment. This cultivation-independent technique is standardly employed in microbial ecology and related fields to study microorganisms, including their genetic diversity and functional potential [1]. A decrease in high throughput sequencing costs has resulted in an exponential increase in metagenome data available [2]. However, only a few repositories provide permanent storage and public access to metagenome data. One of the major repositories is the Sequence Read Archive (SRA) [3]. The SRA is part of the international nucleotide sequence database collaboration (INSDC) [4] between the National Center for Biotechnology Institute (NCBI) [5], DNA Databank of Japan (DDBJ) [6], and European Nucleotide Archive (ENA) [7]. Other repositories, similar to SRA, include Metagenomics Rapid Annotation using Subsystems Technology (MG-RAST) [8], MGnify [9], and gcMeta [10].

The databases allow for the reusability of deposited sequence data for re-analyses and meta-analyses. Advances in computational technology and the development of novel bioinformatics tools and pipelines enable the handling and processing of big datasets and operation of large-scale, comparative meta-studies, leading to novel insights and discoveries. It can help address questions requiring analyses across several geographical regions or over significant periods and test new hypotheses that are impossible or very difficult to test on a single dataset from one research group, usually composed of a limited number of samples. For example, a recent study by Nayfach et al*.* [11] recovered metagenome-assembled genomes (MAGs) from more than 10,000 publicly available metagenomes from different terrestrial and marine environments and hosts. Their genomic catalog expanded the known diversity of bacteria and archaea by 44%. A similar study by Parks et al. [12], using more than 1500 public metagenomes, enlarged the phylogenetic diversity of bacterial and archaeal genome trees by over 30%. However, mining metagenomes from public repositories can be daunting due to unavailable, misleading, or incomplete metadata [13]. This fact significantly contributes to the underutilization of publicly available metagenomes by the scientific community.

Efforts to make mining public repositories easier led to initiatives such as the Genomics Standard Consortium [14], BioProject, and BioSample project [15], which define the required minimum information for a given metagenomic sample and facilitate capture and organization of metadata, respectively [16]. These initiatives have improved metadata annotation and accessibility. Recently, domain-specific standardization started to emerge. For example, HumanMetagenomeDB release 1.0 contains standardized metadata of roughly 70,000 human metagenomes [17]. 

TerrestrialMetagenomeDB release 2.0 contains curated and standardized metadata of over 20,000 terrestrial metagenomes [18]. For marine metagenomic samples, Planet Microbe provides a web-based portal that allows access to standardized metadata associated with marine sequencing data along with biological and physicochemical parameters of the sampling environment [19]. However, it contains only sequence data from coordinated oceanographic sequencing efforts, including the Hawaii Ocean Time-series (HOT) [20], Global Ocean Sampling Expedition (GOS) [21], and large-scale ocean initiatives such as Ocean Sampling Day (OSD) [22], TARA Oceans [23] and Global Oceans Viromes 2.0 [24].

In Planet Microbe, for samples to be added to the portal, they need to contain a minimum of seven attributes (i.e., collection date, latitude, longitude, depth, biome, environmental feature, and material). This requirement results in a lower number of metagenomes when compared to public repositories such as SRA, which adapted Minimum Information about any (x) Sequence (MIxS) and MIMARKS to encode their minimal metadata standards at the sample level [16].

More recently, Yoshitake et al. [25] developed the Ocean Monitoring Database for time-series metagenomics data. However, this database is limited to samples collected from a single study conducted on the Northeastern Pacific coast of Japan.

We created the MarineMetagenomeDB to address the limitations of non-standardized and ambiguous metadata on the searchability, contextualization, and ecological interpretation of marine microbial metagenomes. This metadata database focuses on marine metagenomes present in SRA and MG-RAST. Its primary goal is to help scientists researching marine environments find metagenomes of interest that could be compared with novel datasets or used in meta-analysis studies. In addition, our database describes metadata related to biological samples and technical aspects of the sequencing data. By having standardized metadata, we promote FAIR (Findable, Accessible, Interoperable, and Reusable) principles in microbiome research. MarineMetagenomeDB is meant to supplement recent efforts like Planet Microbe [19], and also promote the exploration and comparison of publicly available marine metagenomes in a user-friendly interface.

## Implementation

### Database construction

**Fig. 1:**
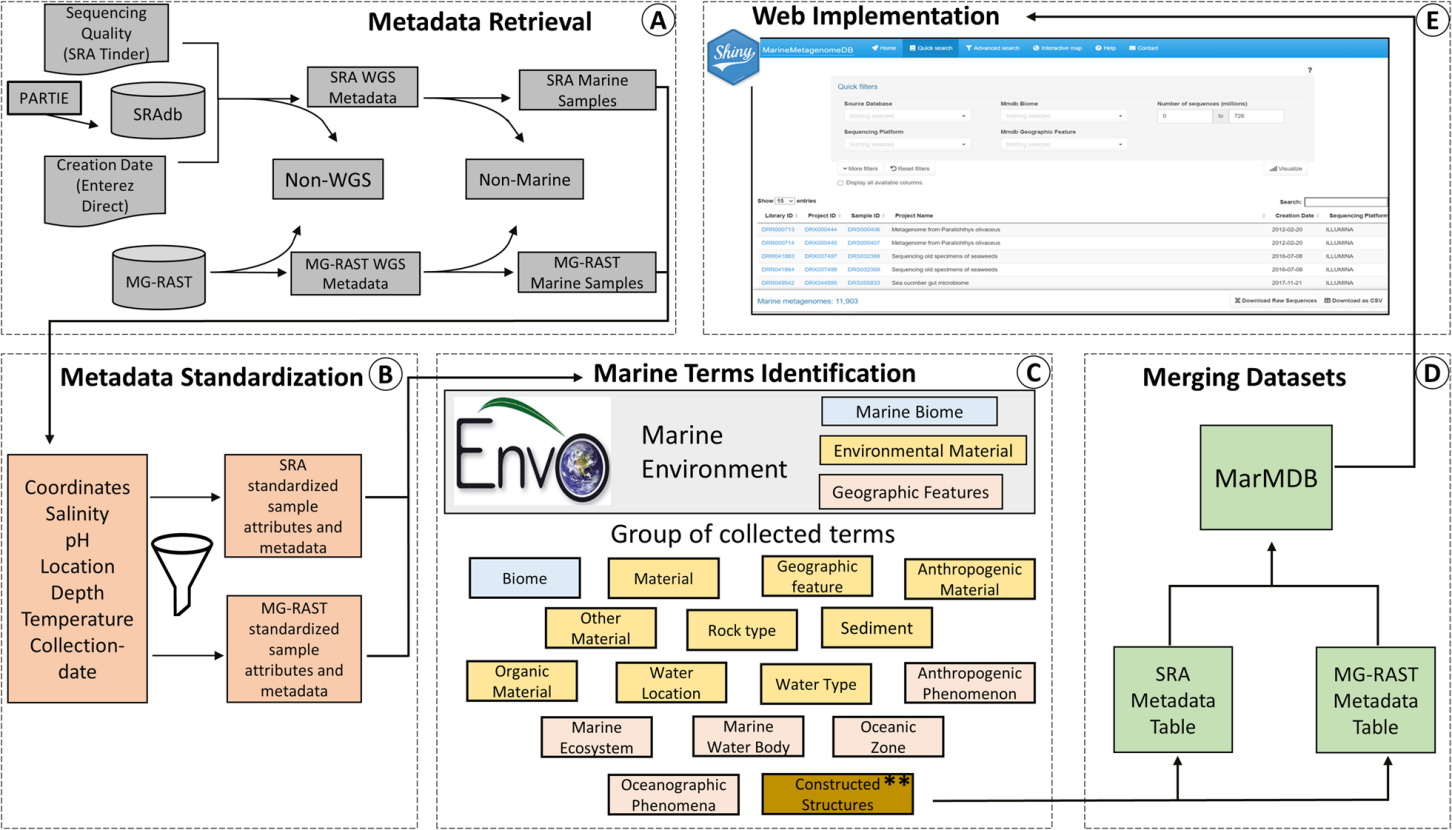
Overview of the MarineMetagenomeDB construction workflow. **A** Metadata retrieval for both SRA and MG-RAST samples was achieved using different pipelines, as explained in the text. Followed by the removal of non-WGS and non-marine samples. **B** Standardization of attributes. Sample attributes including ‘Dates’, ‘Location’ and ‘Depth’ were standardized (**C**) Identification of marine terms. Marine terms were adapted from Marine Biome, Environmental Material, and Geographic Feature of the Environmental Ontology (ENVO). An example of a collection of terms grouped as ‘constructed structures’ (labeled with a double asterisk) can be found in the Additional file (see Additional file 3: Table S3) (‘MarMDB_constructed_structures’) (**D**) Merging SRA and MG-RAST dataset. The MG-RAST attributes were adapted to the SRA metadata standard. **E** MarineMetagenomeDB is made available online through a shiny web implementation. Adapted from [17, 18]

Briefly, we constructed the MarineMetagenomeDB as follows (Fig. 1). First, we retrieved the metagenomes and metadata from the source databases and removed non-marine metagenomes. Next, we parsed and standardized sample attributes of the marine samples. Then, we identified and grouped marine terms. Lastly, we combined the marine metagenomes from the two source databases (SRA and MG-RAST) and implemented the web application. Below we detail the main steps of database construction.

### Metadata retrieval

Similar to TerrestrialMetagenomeDB [18], we selected the two largest repositories (SRA and MG-RAST) of publicly available metagenomes. Metadata from SRA was retrieved using the following: (i) List of sample identifiers (SRA run IDs) labeled as whole-genome sequencing (WGS), or amplicon sequencing was downloaded from PARTIE [13] (https://github.com/linsalrob/partie). PARTIE is a Machine Learning model based on supervised and unsupervised classification, and it was optimized to differentiate sequence read data into WGS and amplicon sequence data sets; (ii) Sample identifiers labeled as WGS were extracted from the list; (iii) Metadata of WGS samples was retrieved using SRAdb R package. This package provides access to metadata of samples available in SRA. (iv) Metadata from the MG-RAST repository was retrieved using their application program interface (API). (v) Furthermore, we retrieved quality scores and the creation date of the SRA libraries using SRA-Tinder (https://github.com/NCBI-Hackathons/SRA_Tinder) and Entrez Direct (https://www.ncbi.nlm.nih.gov/books/NBK179288), respectively. (vi) Finally, we recovered the PubMed and BioProject IDs using rentrez (https://github.com/ropensci/rentrez).

### Removal of non-whole genome sequencing (non-WGS) samples

We removed non-whole genome sequencing (non-WGS) samples retrieved from SRA and MG-RAST. For SRA, non-WGS samples were eliminated by removing samples with the terms ‘AMPLICON’ or ‘*RNA*’ in ‘library_strategy’. Samples marked with ‘*PCR*’ in ‘library_selection’ were also removed. Lastly, all samples marked with anything other than ‘METAGENOMIC’ or ‘GENOMIC’ as their ‘library_source’ were removed. For MG-RAST, all samples marked with any text other than ‘WGS’ as their ‘investigation_type’ and ‘seq_meth’ were removed.

### Identification of metagenomes from marine environments based on metadata

The following strategy was used to select marine metagenomes: (i) using the tool is-sea (https://github.com/simonepri/is-sea), coordinates of the samples were used to classify samples into marine (those with marine coordinates), non-marine (those with coordinates that are not marine) and undefined (those without coordinates); (ii) dictionaries for marine, human, non-marine plant, non-marine animal and terrestrial terms were manually created, respectively (see Additional file 1: Table S1); (iii) the columns ‘center_project_name’, ‘sample_attribute’ and ‘study_title’ were extracted. The columns contain information about the source of the sequence data; (iv) a three-dimensional vector that contained labels ‘keep’, ‘remove’ or ‘NA’ was created for each of the columns (see Additional file 2: Table S2). For each one of the 3 columns, samples were labeled ‘keep’ when they contained marine terms, ‘remove’ when they contained either only non-marine terms, or both, marine and non-marine terms, and ‘NA’ when they contained none of the terms in the dictionaries; (v) samples marked with either only ‘remove’ or ‘remove’ and ‘NA’ were eliminated; (vi) samples marked with either only ‘keep’ or ‘keep’ and ‘NA’ were retained; (vii) samples were classified as ‘check’ when they contained both ‘keep’ and ‘remove’; (viii) samples marked with only ‘NA’s’ were labeled ‘undefined’; (ix) Moreover, we extracted the column ‘study_abstract’ to determine, case by case, whether samples classified as ‘check’ and ‘undefined’ were marine or not. All outputs were manually inspected afterward.

### Standardization of sample attributes

In SRAdb, all sample features are found in a single field named ‘sample_attributes’. Therefore, the features’ names (terms) and values of the samples in the “sample attributes” field are not coherently organized into precise and well-defined metadata categories. We parsed the field attribute names and determined their frequency of occurrence. We removed attribute names with less than 10 occurrences. Next, we grouped synonymous attribute names (see Additional file 3: Table S3). Further, we extracted and standardized the values of eight different attributes: sample collection date, sample salinity, sample latitude, sample longitude, sample depth, sample pH, sample temperature, and sample location (country and sea/ocean). Dates were standardized using International Standard Organization (ISO) 8601 (YYYY-MM-DD). Location (country) was manually labeled following the standard of ISO 3166-1. Sample latitude and longitude were standardized to the format of decimal degrees. A set of terms related to ‘Marine biome’, ‘Environmental Material’ and ‘Geographic Feature’ were adapted from The Environment Ontology (ENVO) [26]. Additional terms associated with constructed marine structures (manually curated by our team) were added to the set of ENVO adapted terms. The set of terms were queried against the metadata to obtain the relevant marine terms in the metadata. The relevant terms obtained were categorized into 16 marine groups (see Additional file 4: Table S4). The terms were further queried against the metadata and assigned samples to their respective marine groups. The set of standardized attributes can be found in the additional files (see Additional file 5: Table S5).

### Merging SRA and MG-RAST metadata files

We identified equivalent and comparable attributes from the curated SRA metadata with those in the metadata provided by MG-RAST (see Additional file 6: Table S6). In addition, the two metadata tables were merged. Five attributes (three related to library sequencing quality and two to sample attributes) were specific and unique to SRA and MG-RAST, respectively. They are; ‘quality_above_30_SRA’, ‘mean_quality_SRA’, ‘sample_pH’, ‘sample_salinity’ for SRA, and ‘drisee_score_raw_MGRAST’ for MG-RAST.

### MarineMetagenomeDB Web app implementation

The MarineMetagenomeDB web application was implemented using Shiny (version 1.5.0), which is an R package used to facilitate the building of an interactive web applications (R version 3.6.3). The app was designed with a tab layout. The tabs include ‘Home’, ‘Quick search’, ‘Advanced search’, ‘Interactive map’, ‘Help’, and ‘Contact’. The ‘Home’ tab steers users around the application. The tab ‘Quick search’ and ‘Advanced search’ provide filter options to aid users select samples of interest. The ‘Interactive map’ tab allows users to select samples based on location. The interactive map functionality was implemented using the leaflet package (version 2.0.3). The selection toolbox for selecting areas on the map was implemented with the geoshaper package (version 0.1.0) and the sp package (version 1.4-2). The remaining packages and their respective versions can be found in the additional files (see Additional file 7: Table S7). The web application is available at https://webapp.ufz.de/marmdb/.

## Results

### Database content

**Fig. 2:**
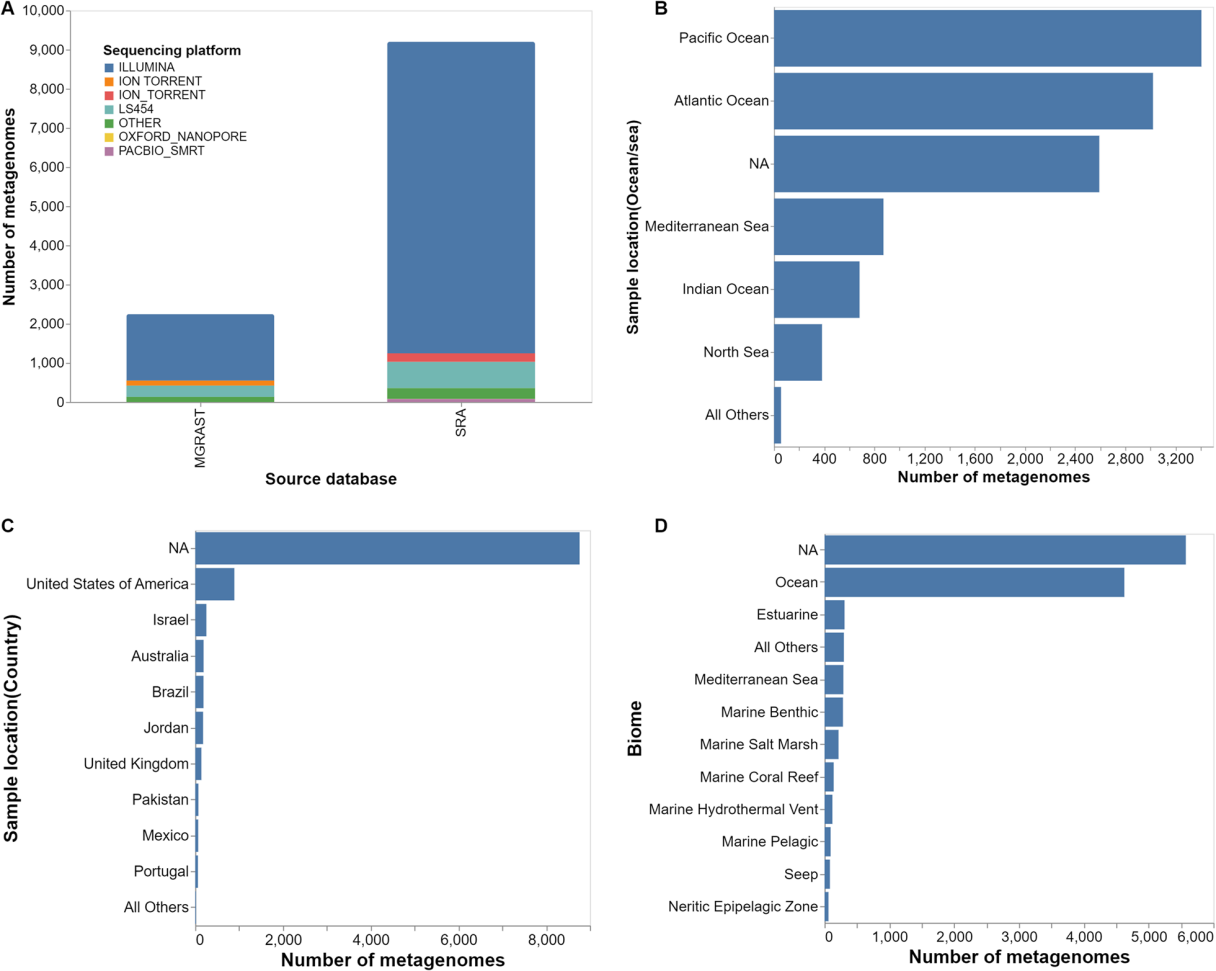
Descriptive statistics of the MarineMetagenomeDB content. **A** Bar plot of the distribution of sequencing technologies (Sequencing platform) per database of origin (Source database). **B** Bar plot of the distribution of the top ten (10) sequencing countries of origin of the metagenomic samples (Sample location). **C** Bar plot of the distribution of the top ten (10) sequencing water bodies (oceans/sea) where the metagenomic samples were collected. **D** Bar plot of the distribution of the top 10 biomes where the metagenomic samples were collected

**Fig. 3:**
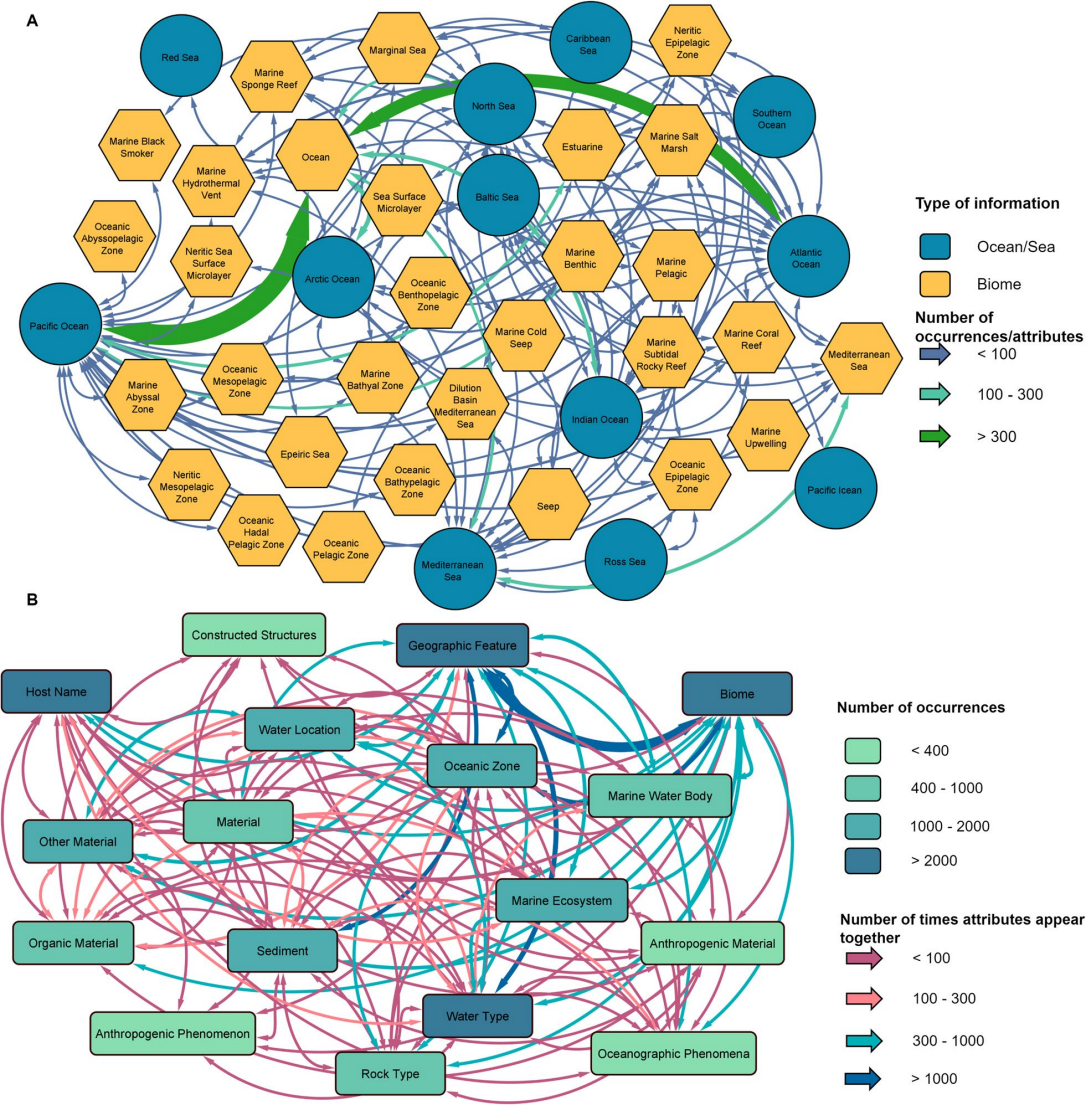
Co-occurrence of the MarineMetagenomeDB attributes. **A** Network representation of the frequencies of biomes and water bodies (Ocean/sea). **B** Network representation of frequencies of marine attributes. For all network graphs, not assigned (NA) values were omitted

The current release 1.0 of MarineMetagenomeDB contains metadata of 11,449 marine metagenomes. Of these, 9202 (80.37%) samples originated from SRA, and the remaining 2247 (19.63%) were obtained from MG-RAST. The database contains experiments submitted over 13 years (from 2007 to 2020). Illumina was the most frequently used sequencing technology 9655 samples (84.33%), followed by LS454, used in 962 samples (8.40%), and ion torrent, used in 216 samples (1.87%) (Fig. 2A). The Pacific Ocean, with 3406 (29.75%), and the Atlantic Ocean, with 3020 (26.38%) represent the water bodies with the highest number of samples. Other water bodies such as the Indian Ocean and the Mediterranean Sea account for 872 (7.62%) and 681 (5.95%) of the samples, respectively (Fig. 2B). For samples collected from water bodies within boundaries of countries, the United States of America contributed the highest number of samples with 891 (7.78%), followed by Israel, Australia, and Brazil with 253 (2.21%), 189 (1.65%), and 187 (1.63%) samples, respectively (Fig. 2C). Strikingly, about 50% of the samples carried no information related to their associated biome. For samples with biome information, the term “ocean” had the highest number of occurrences, with 4624 (40.39%). In comparison, “estuarine” and “marine benthic” were the second and third most abundant biomes with a frequency of occurrence of 308 (2.69%) and 290 (2.53%), respectively (Fig. 2D). The co-occurrence network between the biomes and water bodies in Fig. 3A depicts the biomes where samples were collected. Our network may guide the design of sampling expeditions, as it gives an idea of the explored and unexplored parts of our oceans and seas.

The most populated MarineMetagenomeDB attribute is ‘MarMDB_geographic_feature’, with 8125 (70.97%) of the present values annotated in the metadata. Other attributes with more than 10% of their values annotated in the metadata are ‘MarMDB_biome’, ‘MarMDB_water_type’, ‘MarMDB_sediment’, ‘MarMDB_oceanic_zone’, ‘MarMDB_marine_ecosystem’ and ‘MarMDB_other_material’. Some of the marine terms identified in the metadata co-occurred for the same metagenome. The frequency and co-occurrence of all the MarineMetagenomeDB marine attributes were visualized in Fig. 3B. The remaining ten categories of MarineMetagenomeDB attributes defined in this work appeared in a lower frequency, with the least populated being ‘MarMDB_anthropogenic_phenomenon’ with 73 (0.64%) occurrences and ‘MarMDB_man_made_structures’ with 78 (0.68%) occurrences. Additional file 8: Fig. S1 depicts the percentage of missing values per attribute in the current MarineMetafgenomeDB data.

### Usage and functionalities of the web app

**Fig. 4:**
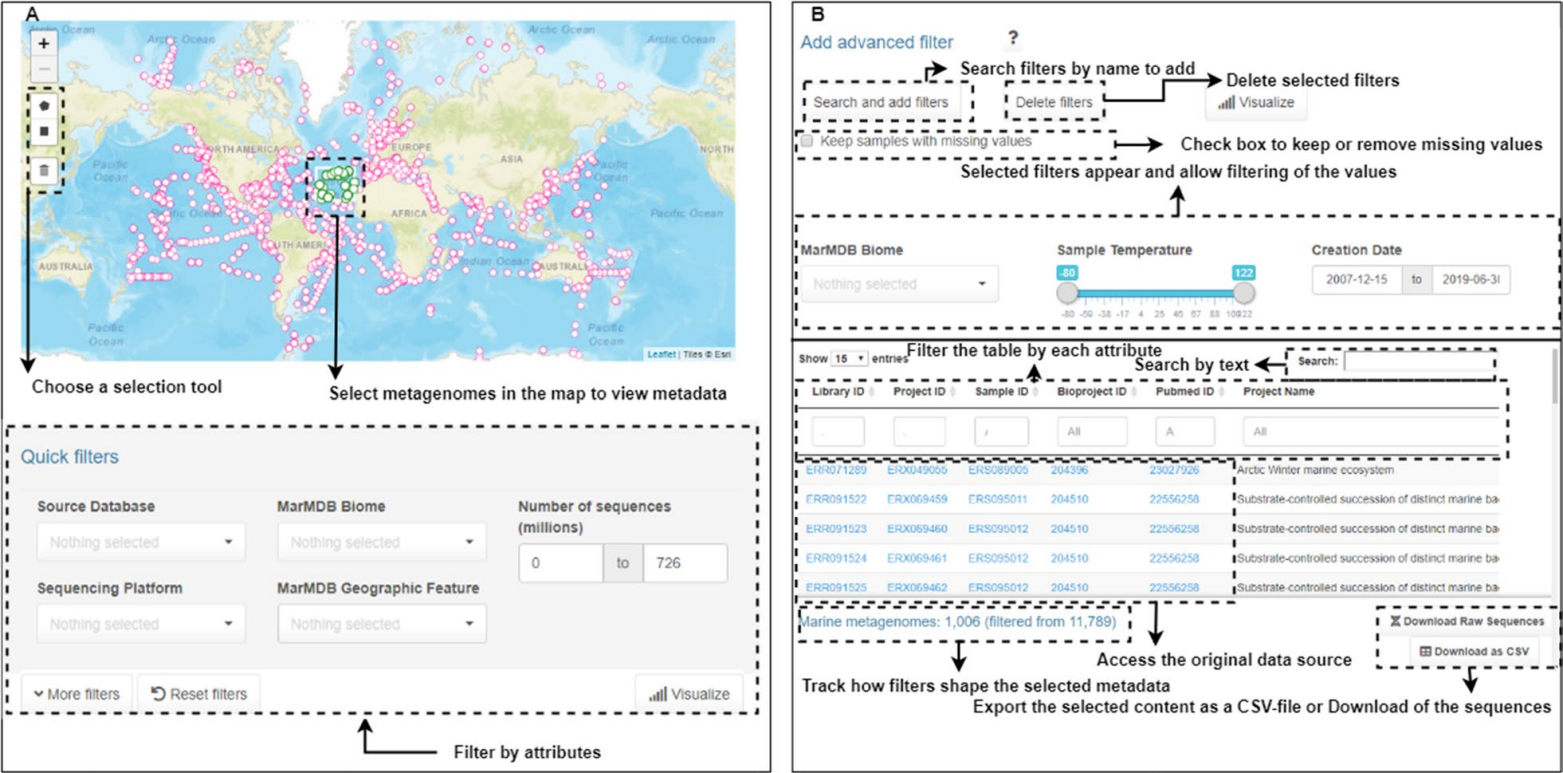
MarineMetagenomeDB user-interface overview. **A** The ‘Interactive Map’ allows users to select samples according to their geographical location on the map using a selection tool, **B** The ‘Advanced search’ tab allows users to select as many filters as they want, and the metadata is displayed under the filtering options

The MarineMetagenomeDB user interface provides easy access to different functionalities to aid in selecting and downloading samples of interest. The user interface has three main sections for users to choose from: ‘Quick Search’, ‘Advanced Search’, and ‘Interactive Map’. Briefly, the ‘Quick Search’ section holds the full content of the current database. Also, it provides options to filter samples by their main characteristics, including Biome, Environmental Material, and Geographic features. The ‘Advanced search’ section contains filters of all attributes in the dataset, allowing users to dynamically search for more specific attributes of interest such as ‘MarMDB Material’, ‘Collection_date’, ‘Assembled’, among others. The ‘Interactive Map’ section provides a graphical method of selecting samples by location directly from the world map. However, it is limited to samples with valid geographical coordinates. Sample identification information (‘sample_id’, ‘project_id’ and ‘library_id’, ‘PubMed ID’ and ‘BioProject ID’) are hyperlinked to the source databases (when available). All tabs include features to visualize the distribution of selected data. Under the ‘Visualize’ button, the user can see a pie chart showing the percentage of the data selected from the complete dataset. An interactive histogram for all available attributes is generated to help users better understand the selected data distribution. A summary table for the selected attributes is also available to help users better understand the selected data's distribution in the attribute explored. Figure 4 shows an overview of the MarineMetagenomeDB user interface. Users may find our video tutorials on how to use the MarineMetagenomeDB in the link (https://www.youtube.com/channel/UCZlcoI8xiWno0mD9V954qRA).

### Quick search

The ‘Quick search’ tab provides users access to the complete content of the MarineMetagenomeDB. Here, filtering of the dataset can be achieved based on the main available attributes. All metagenomes, including those without valid coordinates, are shown. Users can filter entries using 30 available filters or by typing in the search box placed at the top (right) of the table. Afterward, metadata of selected entries can be downloaded as a comma-separated values (.csv) file. If the user does not apply any filter, the metadata of the entire dataset can be downloaded. The steps necessary to obtain raw sequencing data are described in the section ‘Downloading raw data from selected metagenomes’.

### Advanced search

The tab ‘Advanced search’, allows the generation of dynamic features of all available attributes in the dataset. A checkbox was implemented to allow users to exclude samples with missing values for the chosen attributes. The user can click on the ‘Search and add filters’ button and open a window. Searches for attributes can be done by name, but they are organized using the following categories: ‘Sample Attributes’, ‘Environmental Material’, ‘Geographic Feature’, ‘Sample Location’, and ‘Sequencing Features’. After selecting filters and associated values, metadata of selected entries can be downloaded as comma-separated values (.csv) files.

### Interactive map

The interactive map allows users to identify samples from locations of interest on a world map. The map displays only those samples with valid coordinates. We implemented drawing tools (rectangular or polygon shapes) to help users select samples on the map. It is important to note that individual points marked on the map may represent more than one sample since multiple samples can come from the same coordinate position. After selecting samples on the map, their respective metadata are shown on the dataset table below the map. Users can then further limit the entries using filters present in the ‘Quick search’ tab or by typing in the search box placed at the top of the table. After filtering, the resulting metadata table can be downloaded as a comma-separated values (.csv) file.

### Downloading raw data from selected metagenomes

We developed a simple download procedure to obtain raw data from SRA. Unfortunately, MG-RAST does not allow public downloads anymore. Our python scripts enable simple installation of the SRAtoolkit (https://trace.ncbi.nlm.nih.gov/Traces/sra/sra.cgi?view=software), and download of the comma-separated values (CSV) exported metagenomes using two user-friendly commands. To support less experienced users, a script with a graphical user interface (GUI) is available. Although most users may operate on Linux systems, we provide Windows executables to allow instant execution without installation. The download scripts are [AB1] compatible with the CSV exports from the TerrestrialMetagenomeDB [18] and the HumanMetagenomeDB [17] and are provided at https://github.com/mdsufz/downloadtool.

### Usage example

The scientific community interested in finding differences in metagenomes of estuary biomes of different countries may use the MarineMetagenomeDB to find the samples needed to answer this question. On the quick search tab, under ‘Quick filters’, the user can search for estuarine under ‘MarMDB Biome’, resulting in a list of 266 samples. The user can use the ‘More filters’ tab to select samples from countries of interest under the ‘Sample Location Country’ filter. After, the user may select samples, for example, ‘United States of America’ and ‘Australia’, decreasing the number of samples to 66. At this stage, the user can click ‘Visualize’ to explore the selection. A simple exploration of the metadata shows that the “water type” where the samples were collected was either determined as “brackish water”, “saline water”, “sea water” or “NA”. Finally, the user can download the selected metadata dataset as a CSV file for further analysis and use our provided tool to download the raw sequence data of the selected samples.

### Database update plan

As the number of metagenomic experiments submitted to public repositories (e.g., SRA) is continuously rising, we plan to update the database with newly submitted samples once every year in February. Moreover, new features could be added or existing features modified at any time if justified. New unique entries and metadata types will be analyzed and categorized appropriately. The website server will be supported by the German National research Data Infrastructure (https://www.nfdi.de/?lang=en); more specifically, the NFDI4Microbiota consortium (https://nfdi4microbiota.de/). The vision of the NFDI4Microbiota consortium is that researchers in microbiology (including bacteriology, virology, protistology, mycology, and parasitology) can translate research data easily into a deep understanding of microbial species and their interactions on a molecular level. Using the support from the NFDI4Microbiota consortium, the MarineMetagenomeDB will be updated yearly (i.e., every February) to help users to easily find and download metadata and raw data of marine-related samples.

### Suggestions for good practices

One of the goals of this work was to provide ontologies that may facilitate meta-analyses using marine metagenomes present in the largest public repositories (i.e., SRA and MG-RAST). To achieve this goal, we combined known ontologies terms from ENVO with manually curated terms and categorized them into groups of related terms. Further, our effort is integrated into the NFDI4Microbiota that may use the dictionaries developed for the MarineMetagenomeDB as an international reference for marine ontologies. To this end, we included a guide to help the scientific community to annotate their metadata better when submitting novel metagenome samples to public repositories. Suggested ontologies can be located under Point 7 in the ‘Help’ tab of the MarineMetagenomeDB website under the title ‘What should I do to include my metagenomes in MarineMetagenomeDB?’.

## Conclusion

The MarineMetagenomeDB attempts to centralize and standardize marine metagenome samples and their associated metadata available in the Sequence Read Archive (SRA) and MG-RAST. We organized marine terms related to marine biome, environmental material, and geographic features derived from Environment Ontology (ENVO) with the help of the scientific community from different fields of marine research. We manually curated terms associated with constructed marine environments and identified the terms (including ENVO-derived terms) in both SRA and MG-RAST. The MarineMetagenomeDB is in its 1.0.0 release. Due to the rapid increase in novel metagenomes submitted to public repositories, our team will update the MarineMetagenomeDB once every year. This database lays the groundwork for improving the annotation of marine metagenome metadata and facilitates querying and interpretation of marine metagenome samples. In the future, different features could be implemented to improve the MarineMetagenomeDB WebApp. For example, we are working on: (i) how to help users to populate and submit their metadata using our WebApp, (ii) the development of more informative graphics on the home page about selected metadata; and, (iii) creating an API to facilitate the download of raw data and metadata of samples in the database.

## Availability and requirements

- *Project name*: MarineMetagenomeDB.
- *Project home page*: https://webapp.ufz.de/marmdb/
- *Operating system(s)*: Platform independent.
- *Programming language*: R, Python.
- *Other requirements*: Python3.
- *License*: GNU GPL v3.
- *Any restrictions to use by non-academics*: See License.

## Supplementary Information

**Additional file 1**. **Table S1**: Dictionary of terms used for filtering out non-marine samples.

**Additional file 2**. **Table S2**: Some samples with the extracted columns and their labels.

**Additional file 3**. **Table S3**: Attributes chosen to represent different synonyms from SRAdb’s ‘sample_attribute’.

**Additional file 4**. **Table S4**: Marine ‘MarMDB’ attributes groups and their respective values.

**Additional file 5**. **Table S5**: Complete list of attributes in MarineMetagenomeDB. The ‘MarMDB attribute category’ column indicates the manually created category of each attribute.

**Additional file 6**. **Table S6**: Equivalent and comparable attributes in SRA and MG-RAST.

**Additional file 7**. **Table S7**: Packages and their versions used for the WebApp.

**Additional file 8**. **Fig. S1**: Graphic of the percentage of missing values for each attribute of MarineMetagenomeDB by source database.

## Data Availability

The dataset used during the current study is available in the SRA [http://www.ncbi.nlm.nih.gov/Traces/sra] and MG-RAST [http://metagenomics.anl.gov/] repositories.

## Abbreviations

SRA: Sequence read archive
MG-RAST: Metagenomic rapid annotations using subsystems technology
MarineMEtagenomeDB: Marine metagenome database
INSDC: International nucleotide sequence database collaboration
ENA: European nucleotide archive
DDBJ: DNA databank of Japan
MAGs: Metagenome-assembled genomes
HOT: Hawaii Ocean Time-series
GOS: Global ocean sampling expedition
OSD: Ocean sampling day
MIxS: Minimum information about any(x) sequence
MIMARKS: Minimum information about a marker gene sequence
ENVO: Environmental ontology
WGS: Whole genome sequencing
API: Application program interface
ISO: International standard organization
NA: Not assigned
GUI: Graphical user interface
