# Supplementary material for: MarineMetagenomeDB: a public repository for curated and standardized metadata for marine metagenomes
Source: Environ Microbiome. 2022 Nov 18;17:57. doi: 10.1186/s40793-022-00449-7 (PMC9675116; doi:10.1186/s40793-022-00449-7)
Supplement: Supplementary file 1 — Additional file 1. Table S1: Dictionary of terms used for filtering out non-marine samples. [file 40793_2022_449_MOESM1_ESM.pdf]

Table S1. Dictionaries used for filtering out non-marine metagenomic samples

marine_terms	Terrestrial_terms	Plant_and_animal_terms	Human_terms
Anthozoa	Wood	Insect	Human
Pinctada	Root	Equine	Homo
Oyster	Wastewater	Soybean	Human-gut
Hirrondellea	Tundra	Arabidopsis	Man
Oysters	Indoor	Shoot	Sapiens
Coast	Terrestrial	Host-associated	Baby
Atoll	Sewage	Natalensis	Children
Porifera	Lake	Macrotermis	Child
Childressi	Freshwater	Roxelana	Neandertal
Crustacean	Sludge	Albipes	Fetus
Saltmarshes	Forest	Lasioglossum	Infant
Offshore	Air	Seal	Infants
Shore	Soil	Harbor	hmp
Tunicate	Ground	Rubiginosa	Kids
submarine	Humus	Cassida	Kid
Mid-cayman	Dirt	Synthermes	teenagers
Asteroidea	Earth	Wheeleri	
Starfish	Sand	Millet	
Asteroidea-associated	Mineral	Taurus	
Waddenseaa	Land	Endophyte	
Estuarine	Onshore	Rumen	
Estuary	Plastic	Lettuce	
Octocoral	Metal	Pine	
Arctic	Grassland	kakapo	
Seamounts	Mud	Harmonia	
Subseafloor	Rainforest	Sawflies	
Deep-sea	woodland	Chrysochromulina	
Seamount		Baboon	
Beach		Buffalo	
Seawater		Bat	
Intertidal		Tomatoes	
Foreshore		Panda	
Mesoplagic		Lavender	
Pacific		Pollen	
Kelp		Calf	
Vent		Tick	
Vents		termite	
Coastal		Plant birds	
Bathymodiolus		Housefly	
Demersal		Honey	
Hexacoral		Goat	
Gorgonian		Tree	
Epipelagic		Trees	
Epeiric		Elephant	
Deep-sea		Swine	

Abyssopelagic	Invertebrate
Mesopelagic	At
Pelagic	Dog
Acropora	pig
Mediterranean	Hamster
Marine	Rabbit
Ocean	Sheep
Sea	Farm
Atlantic	Mouse
Pacific	Mice
Gulf	Gorilla
Coral	Gallus
Lagoon	Bovine
Bay	Rat
Plankt	Cannis
Permafrost	Chicken
Aquatic	Rattus
Reef	Horse
Mussel	
Trench	
