# Supplementary figures and images for: MarineMetagenomeDB: a public repository for curated and standardized metadata for marine metagenomes
Source: Environ Microbiome. 2022 Nov 18;17:57. doi: 10.1186/s40793-022-00449-7 (PMC9675116; doi:10.1186/s40793-022-00449-7)

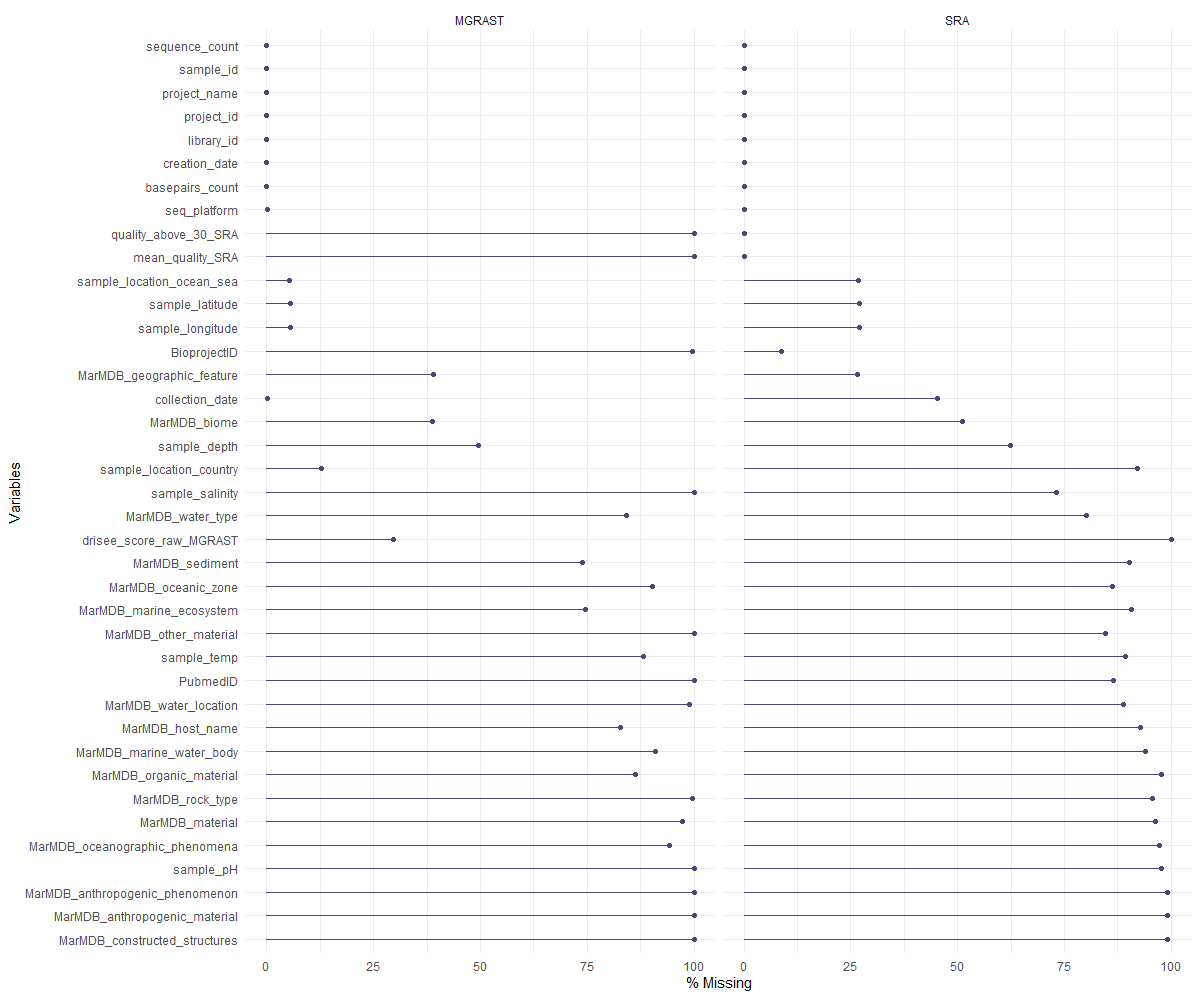

Supplement: Supplementary file 8 — Additional file 8. Fig. S1: Graphic of the percentage of missing values for each attribute of MarineMetagenomeDB by source database. [file 40793_2022_449_MOESM8_ESM.tiff]
